# Formulation, Characterization, Antibacterial Activity, Antioxidant Activity, and Safety Evaluation of *Camphora longepaniculata* Essential Oil Nanoemulsions Through High-Pressure Homogenization

**DOI:** 10.3390/antiox14010033

**Published:** 2024-12-30

**Authors:** Yue Yan, Changhe Wei, Xin Liu, Xin Zhao, Shanmei Zhao, Shuai Tong, Guoyou Ren, Qin Wei

**Affiliations:** 1 Faculty of Agriculture, Forestry and Food Engineering, Yibin University, Yibin 644000, China; 2 Panxi Crops Research and Utilization Key Laboratory of Sichuan Province, College of Agricultural Science, Xichang University, Liangshan 615000, China; 3 Sichuan Province Engineering Technology Research Center of Oil Cinnamon, Yibin University, Yibin 644000, China

**Keywords:** *Camphora longepaniculata* essential oil, chemical composition, nanoemulsions, stability, antibacterial, morphological change, antioxidant

## Abstract

The volatility, instability, and water insolubility of *Camphora longepaniculata* essential oil (CLEO) limit its practical applications in the food, pharmaceutical, and cosmetics industries. CLEO nanoemulsions (CLNEs) were formulated and characterized to overcome the aforementioned issues. The volatile compounds of CLEO were identified by gas chromatography–mass spectrometry. CLNEs were prepared using EL-40 (5%, *w*/*w*) as the surfactant via the high-pressure homogenization method. The formation of nanoemulsions was verified by Fourier transform infrared spectroscopy and transmission electron microscopy. Homogenized nanoemulsions had smaller particle sizes of 39.99 ± 0.47 nm and exhibited enhanced stability. The nanostructured CLEO showed an antibacterial effect against *Escherichia coli* and *Staphylococcus aureus*. The antibacterial mechanism was explored through bacterial morphology and intracellular lysate leakage. CLNEs disrupted the structure of bacterial cells and impaired the permeability of cell membranes, resulting in the leakage of bacterial intracellular contents. The nanoemulsions exhibited superior radical scavenging ability compared to the pure oil. Furthermore, evaluations of the cellular safety of the CLNEs demonstrated that the survival rate of exposed HOECs was greater than 90%, with an apoptosis rate of less than 10% in a concentration range. The results demonstrated that nanoemulsification improved the stability, solubility in aqueous media, and bioavailability of CLEO, thereby broadening its potential industrial applications as a natural antibacterial and antioxidant agent.

## 1. Introduction

Chemically synthesized antioxidants and preservatives have been extensively utilized in the food and pharmaceutical industries. However, these chemicals are potentially toxic to human bodies. For instance, sodium dehydroacetate may induce allergic contact dermatitis and coagulation aberration [1]. Antibiotics are a common pharmacological approach for the treatment of bacterial infections. The issue of antibiotic resistance arising from the misuse of antibiotics is a global public health concern [2]. With the heightened awareness of health and safety in consumption, natural and green chemical preservatives have emerged as a development trend, serving as alternatives to synthetic antimicrobials. The abundant natural products present in plants constitute the primary source of novel antimicrobial substances.

Essential oils are secondary metabolites in plants, characterized by the intense fragrant aroma. These substances have gained considerable attention due to various antimicrobial activities, insecticidal effect, as well as other therapeutic properties. Consequently, they have potential applications in potential applications in flavored, cosmetic, fragrance, or food additive products [3,4].

Despite being recognized as natural preservatives, essential oils often have highly volatile water insolubility and low stability, leading to uneven distribution, rapid depletion, and loss of bioactive potential of antioxidant and antimicrobial activities within products [5]. To overcome the aforementioned issues, nanoemulsions have been introduced as an efficient strategy to preserve and deliver essential oils. Nanoemulsions are colloidal dispersions with droplet sizes typically ranging from 20 to 200 nanometers [6]. Compared to traditional emulsions, nanoemulsions have long kinetic stability. The droplets in nanoemulsions are very small, affording them resistance to particle aggregation or dispersion by gravity [7]. The reduction in droplet size of nanoemulsions is accompanied by an increase in surface area, thereby enhancing their antioxidant and antibacterial properties. Nanoemulsions formed by essential oils can thus exhibit superior performance, improving water solubility, stability, and biological activity [8]. Emulsifiers play a crucial role in the efficiency and durability of nanoemulsions by influencing various factors such as particle size, interfacial tension, viscosity, and particle charge [9]. Common emulsifiers include lecithin, polyethylene glycol castor oil derivatives, and polysorbates [10,11,12]. It is necessary to design nanoemulsion systems containing essential oil components with specific formulations and investigate their antibacterial and antioxidant activities.

The preparation methods of nanoemulsions can be broadly classified into low-energy emulsification and high-energy emulsification approaches. High-pressure homogenization (HPH) is a physical non-thermal high-energy emulsification technology with the advantages of considerable efficiency, low cost, and suitability for industrial production [13]. The principle underlying HPH is that larger droplets are fragmented into smaller ones due to the intense forces of strong turbulence, cavitation, and hydraulic shear when the fluid passes through a constricted aperture [14]. Nanoemulsions fabricated via HPH demonstrated notably small particle sizes with a uniform distribution, along with exceptional stability against centrifugation, pH variations, and thermal challenges [15].

*Camphora longepaniculata* (*C. longepaniculata*) is a local economic tree species native to China, with its primary production area located in Sichuan Province [16]. *C. longepaniculata* essential oil (CLEO) is abundant in leaves and branchlets. Several studies have proven that CLEO possesses antibacterial, antifungal, antioxidant, anticancer, analgesic, and anxiolytic properties [17,18,19]. After being isolated, the main components of CLEO, such as 1,8-cineole (eucalyptol), α-terpineol, terpinen-4-ol, and α-pinene, are widely used in the food, daily chemical, and pharmaceutical industries. 1,8-cineole was used to slow the growth rate of foodborne pathogenic bacteria in meat and extend shelf-life [20]. α-terpineol exhibits antifungal effects against *Aspergillus niger* and *Aspergillus flavus* by inducing morphous damage or metabolic changes [21,22]. Terpinen-4-ol can inhibit biofilm formation both in bacteria and fungi [23,24]. Based on analysis of the efficacy of its primary components, CLEO can be developed as a novel preservative to improve shelf-life and control the risk of pathogenic microorganism-mediated disease contamination.

Some components of essential oils may produce adverse effects at specific dosages. α-Terpinene exhibited neurotoxicity and altered the activity of Na^+^, K^+^-ATPase, and NTPDase in rats when treated with certain dosages [25]. The potential side effects of camphor include pulmonary disorders, stomach discomfort, and other central nervous system-related disorders [26]. Therefore, it is necessary to understand the safety of essential oil preparations.

Although the properties of CLEO have been explored, the range of applications is quite limited. To the best of our knowledge, the creation of nanoemulsions containing CLEO has not been explored in the literature. In this study, CLEO nanoemulsions (CLNEs) were formulated using HPH and subsequently characterized. The antibacterial and antioxidant properties of both the pure oils and the nanoemulsions were evaluated. Additionally, the safety profile of the CLNEs was assessed through cytotoxicity and apoptosis assessments on human normal oral epithelial cells (HOECs). The research findings provide new perspectives for the application of CLEO in a broader range of fields.

## 2. Materials and Methods

### 2.1. Materials

CLEO was provided by Shiping Spice Co., Ltd. (Yibin, China). Castor oil polyoxyethylene EL-40 was purchased from Usolf Chemical Technology Co., Ltd. (Linyi, China). 2,2-diphenyl-1-plcrylhydrazyl (DPPH), 2,2′-azino-bis(3-ethylbenzothiazoline-6-sulfonic acid) (ABTS) was purchased from Aladdin Scientific Corp. (Shanghai, China). Alkaline phosphatase (AKP) and magnesium ions kits were obtained from Jiancheng Bioengineering Institute (Nanjing, China). Cell Counting Kit-8 (CCK-8) kit was purchased from Biosharp life sciences (Hefei, China). Annexin V–APC/PI apoptosis detection kit was obtained from KeyGEN BioTECH Co., Ltd. (Nanjing, China). Other chemical reagents used in this experiment were of analytical or HPLC grade as needed.

### 2.2. Gas Chromatography–Mass Spectrometry (GC-MS) Analysis

The GC-MS analysis was performed in an Agilent 7890 gas chromatograph coupled to an Agilent 5975C mass-selective detector (Agilent Technologies, Santa Clara, CA, USA). A capillary column DB-WAX UI of 60 m × 0.25 mm × 0.25 µm was applied for the separation. Highly pure helium was used as the carrier gas with a flow rate of 1 mL/min. The inlet temperature was set at 270 °C. 0.3 µL of each sample was injected under the split mode with a split ratio at 1:20. The column oven temperature program was initially set at 50 °C and held for 5 min, then the temperature was ramped up at a rate of 10 °C/min to 250 °C and maintained for 20 min.

In MS analysis, electron impact (EI) with an ionization energy of 70 eV was selected as the ionization method. The ion source temperature was set at 250 °C. The interface temperature and the transfer line temperature were 280 °C. The mass spectra were acquired in the full scan mode in the range of 30 to 500 *m*/*z*.

The retention index (RI) for each unknown component was calculated concerning homologous series of C7–C40 saturated alkane mixture using the Van der Dool and Kratz equation. The identification of compounds was carried out by comparing the mass spectra and RI of the known component in the NIST library and other published literature.

### 2.3. Nanoemulsions Preparation

A mixture of CLEO (5%) and EL-40 (5%) at ratios of 1:1 (*w*/*w*) was stirred using a magnetic stirrer with 600 r/min for 15 min. Deionized water was added to the oil phase at a flow rate of 8 mL/min and stirred continuously for 30 min to obtain low-energy emulsified CLNE (unhomogenized CLNE).

The aforementioned nanoemulsions were further homogenized by using a high-pressure homogenizer (AH100D, ATS-Engineering Inc., Brampton, ON, Canada) at 900 bars for 5 cycles to produce a high-energy emulsified CLNE (homogenized CLNE) and then cooled in an ice water bath.

### 2.4. Droplet Sizes and pH of CLNE

CLNE was diluted with deionized water, and then the mean droplet sizes and polydispersity index (PDI) were measured by dynamic light scattering (DLS) technique on a particle size analyzer (Zetasizer Nano ZS90, Malvern Instruments Ltd., Worcester, UK). Based on the particle size results, nanoemulsions with smaller particle sizes were selected for subsequent activity evaluation. The pH of nanoemulsions was measured by an acidity meter (PHS-3C^+^, Century Fangzhou Technology CO., Ltd., Chengdu, China).

### 2.5. Stability Analysis

Freshly prepared 10 mL of CLNE was transferred into a centrifuge tube and centrifuged at 2000× *g*, 4000× *g*, and 6000× *g* for 30 min in a high-speed centrifuge, respectively. The appearance of the nanoemulsions was observed before and after centrifugation.

### 2.6. Transmission Electron Microscopy (TEM) Observation

The CLNE was dropped on the supporting film of a copper mesh, and 2% phosphotungstic acid dye was added. After absorbing the excess dye, the samples were subsequently observed under a transmission electron microscope (JEM2100F, JEOL, Tokyo, Japan).

### 2.7. Fourier Transform Infrared (FTIR) Analysis

The FTIR spectra of CLEO, EL-40, unhomogenized CLNE, and homogenized CLNE were carried out on a spectrophotometer (Nicolet iS50, Thermo Fisher Scientific Inc., Waltham, MA, USA). The spectra were acquired in the range of 400–4000 cm^−1^ with a 4 cm^−1^ resolution for 32 scans.

### 2.8. Antibacterial Activity

#### 2.8.1. Determination of Minimum Inhibitory Concentration (MIC)

The determination of the antibacterial rate of CLEO and CLNE was evaluated against *Escherichia coli* (ATCC 25922) and *Staphylococcus aureus* (CMCC 26003). To disperse the essential oil in an aqueous solution for dilution, a specific concentration of CLEO was added to the aqueous solution and subjected to high-speed shearing at 10,000 rpm for 3 min.

MIC analysis was carried out by the broth dilution method using 96-well microplates. Each well in the plate was inoculated with diluted bacterial strain suspension at a final concentration of 10^6^ CFU/mL. CLEO or CLNE was added to corresponding wells to reach final concentrations ranging from 0.5 to 16 mg/mL. EL-40 solution in the same concentration was also determined for MIC. The positive control wells were equipped with bacterial suspension in LB broth and the negative control wells were prepared with LB broth. The plates were incubated at 37 °C for 24 h. MIC was defined as the lowest concentration of CLNE with no visible bacterial growth in the broth. The MIC of the antimicrobial drug ampicillin was also determined as a reference. For detecting minimum bactericidal concentration (MBC), 100 μL of incubated bacterial broth from wells that contained no visible growth was plated onto LB agar at 37 °C for 24 h. The minimum concentration that resulted in no bacterial colonies on the agar was recorded as MBC. The experiment was repeated three times.

#### 2.8.2. Time–Kill Curve

The bacterial suspension was approximately diluted to 10^6^ CFU/mL in LB broth. CLNE was added to the form final concentrations of 1/2MIC, 1MIC, and 2MIC; 1 mL bacterial solution was added to 100 mL LB medium and incubated at 37 °C. The OD value at 600 nm was measured every 2 h using a spectrophotometer (Macy UV-1500PC, Shanghai, China).

### 2.9. Antibacterial Mechanism

#### 2.9.1. Leakage of AKP and Magnesium Ions

The integrity of the cell wall and membrane was evaluated by measuring the release of intracellular substances (enzymes and ions) into the external environment. Bacteria were incubated in LB broth at 37 °C. Subsequently, the bacterial cells were collected by centrifugation and resuspended in PBS. CLNE was added to form final concentrations of 1/2MIC, 1MIC, and 2MIC and shaken for 5 h. Samples were centrifuged at 5000× *g* for 5 min and suspensions were collected. Alkaline phosphatase and magnesium ions contents in the supernatant were measured using the AKP assay kit and magnesium ions assay kit, following the kits’ instructions.

#### 2.9.2. Scanning Electron Microscope (SEM) Observations

The bacterial suspension was obtained referencing the methodology in the preceding section. Samples were treated with CLNE at the concentrations of MIC for 2 h, and then the supernatant was removed by centrifugation. The cells were immersed in 2.5% glutaraldehyde at 4 °C overnight. The bacteria were dehydrated with gradient concentrations of ethanol at 30%, 50%, 70%, 80%, 90%, and 100%. After drying and gold coating, the samples were sprayed with gold and observed under an SEM (JSM-7800F, JEOL, Tokyo, Japan) to assess cell morphology.

### 2.10. Determination of Antioxidant Activity

#### 2.10.1. DPPH Assay

The antioxidant activities of CLEO, CLNE, and vitamin C were determined using the DPPH radical scavenging method [27,28]. DPPH was dissolved in ethanol and prepared as a 0.1 mmol/L solution. A series of essential oil or nanoemulsion samples with different concentrations were mixed with 1 mL DPPH solution in equal volumes and reacted at room temperature. One milliliter of ethanol was reacted with one milliliter of DPPH solution as a blank control. After reacting in the dark for 30 min, the absorbance was measured immediately at 517 nm. The percentage of scavenging DPPH free radical was calculated by the following equation:

(1) $$\mathrm{DPPH}\;\mathrm{radical}\;\mathrm{scavenging}\;\mathrm{percent}\;(\%)=\frac{A_{0}-A_{1}}{A_{0}}\times 100$$

where A_0_ represents the absorbance of the blank control and A_1_ is the absorbance of the test solution.

#### 2.10.2. ABTS Assay

The scavenging activity against ABTS^•+^ was performed based on a procedure described by Song et al. with some modifications [29]. The ABTS^•+^ reagent was prepared by mixing 7.4 mM ABTS solution with 2.6 mM potassium persulfate. The mixture was kept in the dark at room temperature for 16 h to generate free radicals. The absorbance of ABTS^•+^ solution was diluted with absolute ethanol and adjusted to 0.7 ± 0.02 at 734 nm. Then, 0.5 mL of various concentrations (ranging from 0.25 to 8 mg/mL) of the CLEO or CLNE was added to the ABTS^•+^ solution (2.5 mL) and shaken vigorously. After incubation in the dark at room temperature for 30 min, the absorbance of the solution was measured at 734 nm. The ABTS^•+^ scavenging percentage was calculated with the following formula:

(2) $${\mathrm{ABTS}}^{•+}\;\mathrm{scavenging}\;\mathrm{percent}\;(\%)=\frac{A_{c}-A_{t}}{A_{c}}\times 100$$

where A_c_ represents the absorbance of the mixture of ethanol and ABTS^•+^ solution and A_t_ corresponds to the absorbance of the mixture of tested sample and ABTS^•+^ solution.

### 2.11. Biological Safety Analysis of CLNE

#### 2.11.1. Cell Viability Assay

HOECs were cultured in RPMI 1640 medium with 10% fetal bovine serum and 1% penicillin–streptomycin solution at 37 °C in a humidified atmosphere of 5% CO_2_. Cells in logarithmic growth were resuspended and plated on a 96-well plate at approximate populations of 5 × 10^3^ per well to adherent growth. CLNE was added to achieve final concentrations of 19.5, 39, 78, 156, and 312 μg/mL, and the cells were treated for 24 h. After treatments, the cells were washed with PBS; 10 μL of CCK-8 reagent was added and incubated at 37 °C for 2 h. The amount of water-soluble formazan dye after the reaction was determined by the absorbance at 450 nm using a microplate reader (ELx800, BioTek Instruments, Inc., Winooski, VT, USA).

#### 2.11.2. Cell Apoptosis

A total of 2 × 10^5^ HOECs were incubated with CLNE at concentrations of 156 and 312 μg/mL for 24 h. Attached cells were digested with trypsin and centrifuged at 250× *g* for 5 min. Then cells were washed with PBS. After centrifugation, the supernatant was discarded, and the cells were resuspended in a binding buffer; 5 μL Annexin V–APC and 5 μL PI were sequentially added and mixed gently, then incubated in the dark for 15 min. The samples were analyzed using a flow cytometer (CytoFLEX, Beckman Coulter, Brea, CA, USA).

### 2.12. Statistical Analysis

The result values were expressed as mean ± standard deviation from three replicate experiments. One-way analysis of variance (ANOVA) with Duncan test and paired *t*-tests were used to determine the significant differences by SPSS 26.0 software. Comparison was made with statistical significance level of *p* < 0.05.

## 3. Results and Discussion

### 3.1. Chemical Composition of CLEO

Through GC-MS analysis, 31 compounds were separated and identified, accounting for approximately 99.1% of the total composition of the essential oil (Table 1).

Monoterpenes were the predominant in the essential oil, accounting for 97.39%, followed by sesquiterpenes with 1.73%. 1,8-cineole was the major compound in CLEO, constituting 49.49% of the oil content. Other monoterpenes were primarily represented by sabinene (12.11%), α-terpineol (7.7%), α-pinene (7.4%), β-pinene (4.28%), and terpinen-4-ol (3.82%). In sesquiterpenes, bicyclogermacrene (0.96%) and α-humulene (0.49%) were the predominant components.

These findings were similar to those of previous studies. Chen et al. reported that the main compounds of CLEO include 1,8-cineole, α-terpineol, and terpinen-4-ol [30]. However, the composition and percentage of essential oil components are not entirely consistent with other studies. These differences may be attributed to variations in extraction methods, geographical conditions, phenological stages, collection time, and spontaneous out-crossing [31,32]. The CLEO utilized in this study was extracted from the leaves of *C. longepaniculata* using industrial steam distillation. During the extraction process, the plant material remains separated from the water, and the volatile components are distilled out along with the water vapor. It is a type of commercial essential oil, thus possessing a high degree of representativeness.

### 3.2. Characterization of the Nanoemulsions

#### 3.2.1. Mean Particle Size, Size Distribution, and pH

**Figure 1:**
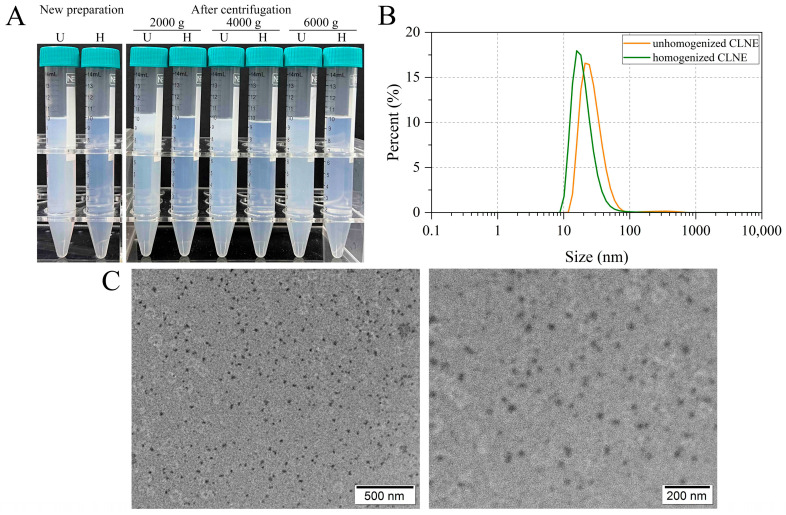
Morphology of nanoemulsions. (**A**) Appearance of nanoemulsions and centrifugal stability. U: unhomogenized CLNE; H: homogenized CLNE. (**B**) Particle size distribution. (**C**) Morphology of homogenized CLNE.

Visually, both unhomogenized and homogenized CLNEs appeared translucent, yet the homogenized exhibited greater transparency (Figure 1A). In the nanoemulsion type, the transparent optical aspect indicates small droplet sizes.

The average droplet sizes of unhomogenized and homogenized CLNEs were 74.90 ± 3.45 nm and 39.99 ± 0.47nm, respectively. The particle size distribution is presented in Figure 1B. The majority of droplets had a diameter smaller than 100 nm. The narrow peak width showed a uniform droplet size distribution, indicating the formation of a homogeneous colloid. The PDI was 0.416 ± 0.004. Excessive oil carrier can lead to aggregation, affecting the formation of nanoemulsions. Thus, an appropriate ratio of essential oil to oil carrier can keep the nanoemulsions in a good state, favoring the reduction in droplet size. Smaller droplet sizes facilitate particle penetration and exhibit good resistance to gravitational dispersion and aggregation. After HPH, nanoemulsions with smaller particle diameters and narrower peak widths can be obtained. This result confirmed the apparent morphology. Therefore, for subsequent experiments, homogenized nanoemulsions were chosen for subsequent investigation.

The pH of CLEO and CLNE were 3.83 ± 0.06 and 5.39 ± 0.03, respectively. The pH of the formed nanoemulsions was closer to neutral, making nanoemulsions suitable for usage.

#### 3.2.2. Stability

Through observation of the appearance of the nanoemulsions before and after centrifugation, it was found that the unhomogenized nanoemulsions exhibited different degrees of stratification after centrifugation at 2000× *g* and 4000× *g*. Similarly, when centrifuged at 6000× *g*, a certain degree of turbidity was also displayed. In contrast, the homogenized nanoemulsions all appeared transparent and clear, showing no difference compared to their state before centrifugation. The results indicate that the nanoemulsions system constructed using CLEO and EL-40 mixed in a 1:1 ratio and emulsified by HPH in this study exhibited stability, with small average particle size and good dispersion of droplets, and no significant visible aggregation observed.

#### 3.2.3. Morphology of Nanoemulsions

The morphology of CLNE was observed under the TEM as shown in Figure 1C. The prepared CLNE appeared as dispersed small droplets, spherical in shape, uniformly distributed, and consistent in size.

#### 3.2.4. FTIR Spectroscopy

**Figure 2:**
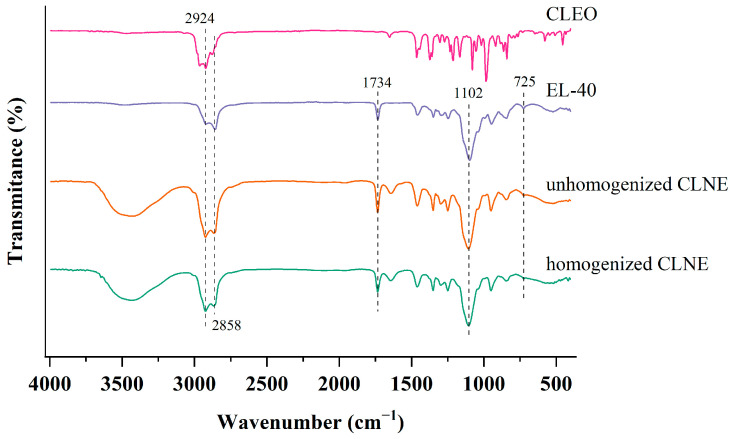
FTIR spectra of CLEO, EL-40, unhomogenized CLNE, and homogenized CLNE.

The FTIR spectra of CLEO, EL-40, unhomogenized CLNE, and homogenized CLNE are presented in Figure 2. The infrared spectrum of CLEO was determined by the main compounds. The peaks at 2965 cm^−1^ and 2924 cm^−1^ corresponded to the stretching vibration of methyl and methylene groups, respectively. Notable peaks reflected the characteristics of 1,8-Cineole: C-C stretching at 1213 cm^−1^, C-O stretching at 1166 cm^−1^, and C-H bending at 1373 cm^−1^ and 1080 cm^−1^ [33]. The weak peak around 3075 cm^−1^ might have been caused by the stretching vibrations of =C–H double bonds from olefins such as sabinene and pinene.

The infrared spectrum of EL-40 presented the characteristics of a long alkyl chain at the peaks of the stretching vibration of methylene at 2924 cm^−1^, 2858 cm^−1^, and the bending vibration at 725 cm^−1^. The carbonyl absorption was observed at 1734 cm^−1^. The bands from 1180 cm^−1^ to 1040 cm^−1^ may be attributed to the stretching vibrations of the C-O-C [34].

The spectrum of the two types of nanoemulsions was generally consistent. The characteristic bands originated from the essential oil and emulsifier [35], except hydroxyl groups stemmed from the aqueous solution. The spectrum that is closer to the emulsifier suggests that CLEO may be encapsulated within EL-40.

### 3.3. Bacteriostatic Activity

#### 3.3.1. MIC and MBC

CLEO has been demonstrated to be inhibitory against common foodborne pathogenic pathogens. CLEO exhibits hydrophobic and volatile characteristics, tending to evaporate from surfaces when applied directly. This evaporation limits its ability to exert antibacterial effects. Therefore, CLEO should be formulated into a form that is easily dispersed in water and reduces volatility. The MIC and MBC values of CLEO and CLNE are presented in Table 2. They were effective against common bacteria, with slight differences. As a control, on the test concentration of EL-40 in this trial, no antibacterial or bactericidal activity was displayed. The antibacterial activity of CLEO originated from its primary components. The range of MIC of 1,8-cineole, α-terpineol, and terpinen-4-ol against *E. coli* and *S. aureus* was 1–8 mg/mL [36,37,38]. The MIC of CLEO against *E. coli* was lower than that of *S. aureus*, demonstrating that *E. coli* is more sensitive to CLEO. To eliminate *E. coli* and *S. aureus*, the concentrations of coarsely dispersed essential oils required are 6 mg and 8 mg, respectively. The MIC and MBC values were slightly higher than that reported in the study by Li et al. [17], indicating optimal results cannot be obtained using coarsely dispersed essential oils.

The nanoemulsion system of encapsulated CLEO enhanced antibacterial activity against two strains, where the MIC and MBC values were less than that of CLEO. These results were similar to previous research about the better inhibitory effect of essential oil nanoemulsions. Lemon essential oil nanoemulsions showed a better inhibitory effect on *S. aureus*, *Klebsiella pneumoniae*, and *Enterococcus faecalis* (MIC values of 3.125–12.5 mg/mL) compared with pure oil (MIC > 25 mg/mL) [39]. The clove essential oil nanoemulsions inhibited the growth of *S. aureus* and *E. coli* at a lower concentration with MIC at 0.65 mg/mL, compared to free essential oil (MIC at 1.3 mg/mL) [35]. The differences in antibacterial activities of nanoemulsions against various bacteria may be attributed to varying sensitivities of the bacteria to essential oils, leading to discrepancies in their effectiveness in inhibiting growth or destroying bacteria. In addition, differences in the type of emulsifier or additive used may also have an impact on the performance of the nanoemulsions. The MIC of ampicillin against *E. coli* and *S. aureus* were 8 and 0.25 μg/mL respectively, lower than that of common essential oils. However, their usage scenarios are different. Antibiotics are mainly used to treat bacterial infections, while essential oils are not the first-line drugs for treating infections. Essential oils are predominantly utilized in cosmetics, daily necessities, and air disinfectants, where antibiotics are not directly applied. Additionally, essential oils have the potential to be applied in combination with antibiotics to reduce the dosage of antibiotics, side effects, and drug resistance. Essential oils of *Ocimum gratissimum*, *Citronella java*, and *Cymbopogon flexuosus* showed synergistic interactions with ampicillin [40].

#### 3.3.2. Effect of CLNE on Bacterial Growth

**Figure 3:**
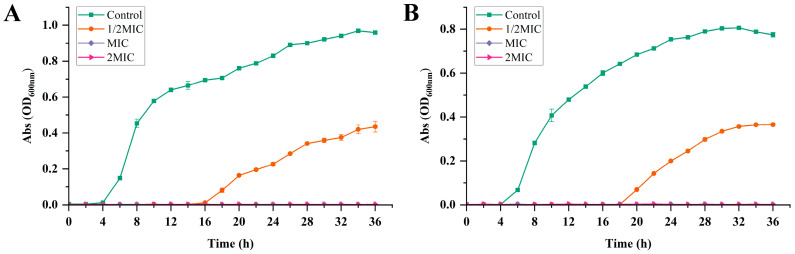
Growth curves of *E. coli* (**A**) and *S. aureus* (**B**) treated with different concentrations of CLNE.

Figure 3 plots the growth curves of bacteria treated with or without CLNE in broth culture at 37 °C for 36 h. As shown in Figure 3A, *E. coli* in the control group entered the logarithmic growth phase from 4 h and sustained continuous growth up to 34 h. Half minimum inhibitory concentration inhibited the growth of *E. coli* for more than 14 h, compared to the control group, with growth initiation observed at 18 h, and the final OD600 value was 0.44. When *E. coli* was treated with CLNE of MIC and 2MIC, the absorbance was not changed obviously. This indicated that the low concentration of CLNE can inhibit *E. coli* for a short time, while CLNE of MIC and 2MIC can suppress *E. coli* for extended periods.

As shown in Figure 3B, *S. aureus* exhibited exponential growth from 4 h in the control group. Upon exposure to 1/2 MIC, no apparent increase in OD value was observed in the first 18 h. At 36 h, the absorbance reached 0.37, lower than the 0.78 observed in the control group. Similarly to *E. coli*, neither the MIC- nor 2MIC-treated group exhibited significant bacterial growth within 36 h. The results demonstrated that CLNEs of different concentrations can delay the bacterial growth cycle to various degrees, and the antibacterial effect appeared to be dose-dependent. Both *E. coli* and *S. aureus* exhibited sensitivity to concentrations of CLNE that exceeded the MIC. This antibacterial trend was similar to other essential oils or their component products against foodborne pathogens [41,42,43].

### 3.4. Antibacterial Mechanisms of CLNE

#### 3.4.1. Effect of CLNE on AKP and Magnesium Ions

**Figure 4:**
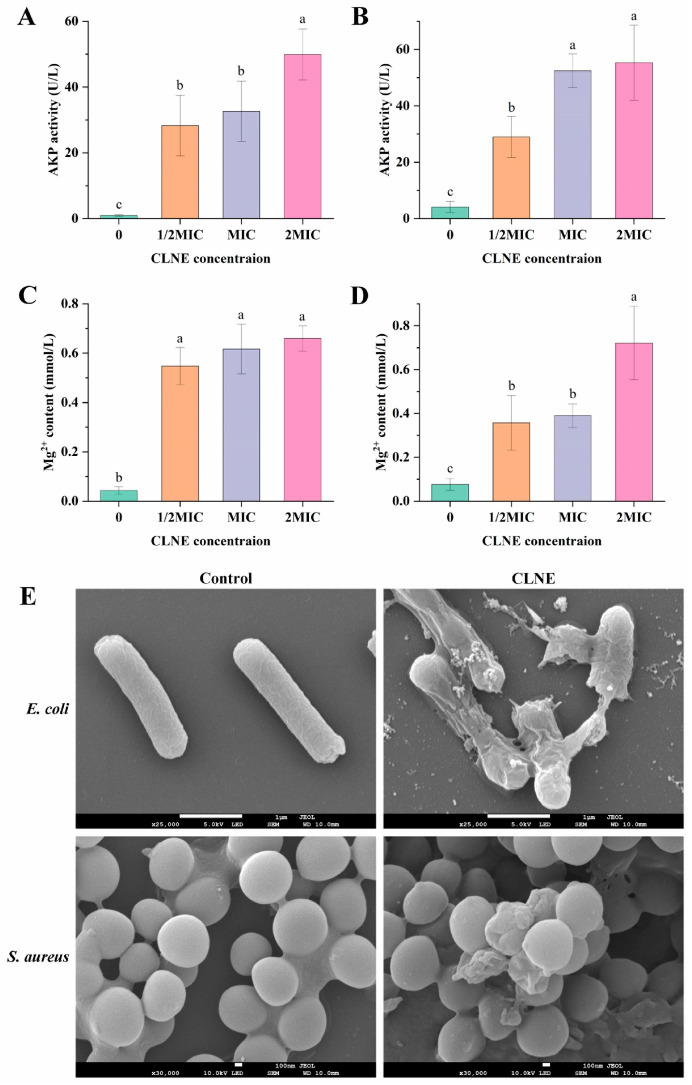
Antibacterial mechanisms of CLNE. (**A**) Release of AKP from *E. coli*; (**B**) release of AKP from *S. aureus*; (**C**) release of Mg^2+^ from *E. coli*; (**D**) release of Mg^2+^ from *S. aureus*; (**E**) SEM photography of *E. coli* and *S. aureus* with CLNE treatments. Diverse lowercase letters (a–c) mean significant differences between groups (*p* < 0.05).

The integrity and permeability of bacterial cell membranes were evaluated in this experiment using AKP and magnesium ions leakage as indicators. AKP is a protease located between the bacterial cell wall and the cell membrane, and it is undetectable in the extracellular environment unless the cell wall is impaired. In this experiment, the AKP activity in the cell suspension was measured to evaluate the damage to the cell wall [3]. According to Figure 4A,B, after the treatment with CLNE at various doses for 5 h, the levels of AKP in *E. coli* and *S. aureus* treated with 1/2MIC, MIC, and 2MIC exceeded 20 U/L, which were higher than that of the control group (*p* < 0.05). The AKP activity in the suspensions of the two strains increased with the rising dosage of CLNE, reaching a maximum of 2MIC. These results suggested that the permeability of the cell wall increased. The cell wall is a rigid protective layer that stabilizes cell shape and withstands osmotic pressure. Additionally, the cell wall effectively prevents the invasion of foreign substances, such as antibiotics and hydrolytic enzymes [44]. The antibacterial effect of CLNE is exerted by disrupting the bacterial cell wall in a short period.

Magnesium ions participate in various metabolic reactions. ATP serves as the primary source of cellular energy and requires the binding of Mg^2+^ to perform biological activity. The complex formed is crucial for the stability of the physiological structure of DNA and RNA [45]. As shown in Figure 4C,D, the Mg^2+^ content in the supernatant of the treated group was significantly higher than that in the control group. CLNE of 2MIC was more efficient in increasing the release of Mg^2+^, compared with that of 1/2MIC and MIC in *S. aureus*. The high release of Mg^2+^ may originate from the increased cell membrane permeability or bacterial death. The trend of magnesium ion leakage was similar to that observed in previous studies on terpinen-4-ol [46]. This indicated that CLNE may have disrupted the cell wall and membrane, ultimately inhibiting bacterial growth.

Previous research has found that the primary antibacterial mechanism of essential oils encompasses increased permeability and plasma membrane disruption. Terpenes and terpenoids containing hydroxyl groups in essential oils can react with the active site of the target enzyme, leading to inactivation [47]. For instance, α-terpineol interacts with the polar head group leading to an increase in gelation and a decrease in membrane fluidity. Concurrently, electron transfer is disrupted, resulting in the accumulation of reactive oxygen species, while the leakage of ATP from the cells exacerbates the energy deficiency [48].

#### 3.4.2. Morphological Changes

Changes in bacterial cell morphology are illustrated in Figure 4E. Untreated *E. coli* exhibited the characteristic rod-shaped morphology, with an intact surface, without any obvious ruptures. In contrast, bacteria treated with CLNE exhibited a rough and wrinkled appearance; some parts appeared to have irregular shapes and were clearly fractured, with the leakage of contents. The morphology of *S. aureus* in control appeared regular in shape, smooth, and round. The CLNE-treated bacteria emerged in irregular and concave shapes, with observable membrane ruptures in partial cells. The presence of CLNE can disrupt membrane integrity, leading to dysfunction in the permeability barrier and ultimately resulting in the loss of cellular viability. These results were consistent with the measured leakage of intracellular contents. This suggested that after treated with CLNE, the membrane structure of the cells sustained continuous damage. Most studies indicate that essential oils can cause cell membrane disruption and cell leakage, although the mechanisms of their main constituents, the terpene compounds, may be different [49].

### 3.5. Antioxidant Activity

**Figure 5:**
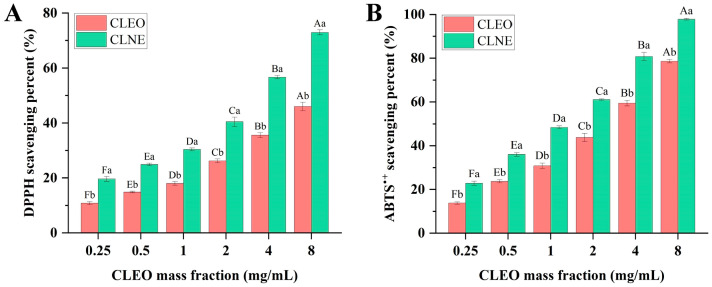
Antioxidant ability of CLEO and CLNE. (**A**) DPPH radical scavenging assay; (**B**) ABTS^•+^ radical scavenging assay. Diverse capital letters indicate significant differences among different concentrations of the same sample, while different lowercase letters indicate a significant difference between the two samples at the same concentration (*p* < 0.05).

The ability to scavenge free radicals is one of the attractive biological properties of natural products. The incorporation of plant essential oils can be used as preservatives to mitigate food oxidation and extend shelf life. As lipophilic antioxidants, essential oils contain a mixture of compounds, that have been utilized in cosmetic preparations to the chain reaction of membrane lipid peroxidation. In this aspect, the antioxidant effects of CLEO and emulsion form were measured by DPPH and ABTS^•+^ free radical scavenging capacities. As shown in Figure 5A, the maximum DPPH scavenging percent of CLNE (72.97% ± 0.92%) was significantly higher than that of CLEO (45.99% ± 1.52%). In terms of ABTS^•+^ scavenging, the maximum clearance rate of CLNE was 97.83% ± 0.41%, which was stronger than that of CLEO (78.63% ± 0.75%) (Figure 5B). The reducing capabilities of both CLEO and CLNE were heightened as their concentrations increased, and a consistent pattern was observed in their scavenging rates for both types of free radicals. In this concentration range, the clearance rate of vitamin C approached 100%. These results indicated that the scavenging rate of CLNE was lower than that of vitamin C. However, CLNE expressed a strong scavenging effect when the mass concentration increased. The antioxidant activity of CLEO may be attributed to the presence of the terpenes. α-Pinene, sabinene, β-pinene, β-myrcene, α-terpinene, and γ-terpinene are the primary reason for the antioxidant potential in different plant essential oils. Additionally, functional groups including phenolic hydroxyl, aldehyde, and alcohol hydroxyl in terpenes can also affect the antioxidant activity of essential oils [50].

At the same concentration, the scavenging rates of the sample for ABTS^•+^ were higher than that for DPPH. This may be due to different mechanisms, where the ABTS^•+^ radical is primarily scavenged by electron transfer, whereas the scavenging of DPPH radicals is mainly associated with hydrogen atom transfer [51]. The scavenging values of CLNE were significantly higher than those of the CLEO at the same mass fraction (*p* < 0.05), demonstrating that nanoemulsions can enhance reducing capacity. The enhancement in antioxidant activity after formulating nanoemulsions may be related to the smaller droplet size and increased specific surface area of essential oils, thereby enabling efficient and rapid adsorption of free radicals. Though antioxidant properties were lower than those of vitamin C, CLEO still exhibited antioxidant activity at certain concentrations, similar to other essential oils [15,52]. The comprehensive efficacy of essential oils has advantages compared to single antioxidants. In general, these results demonstrated that CLNE can serve as accessible natural antioxidant additives in food packaging materials or cosmetics designed to resist oxidation.

### 3.6. Safety Evaluation of CLNE

**Figure 6:**
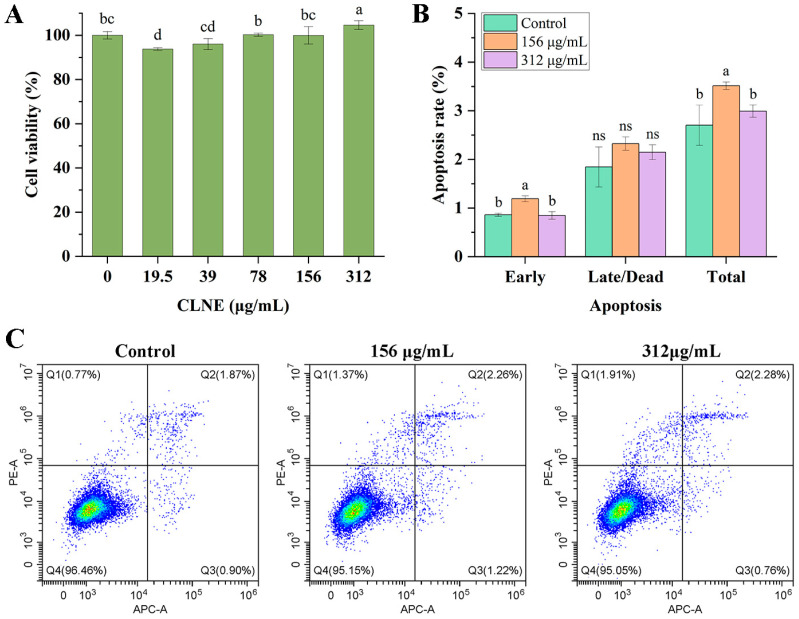
Viability and apoptosis in HOECs treated with CLNE. (**A**) Cell viability with different concentrations of CLNE. (**B**) Percentages of apoptosis. (**C**) Flow cytometry analysis and quantification of apoptotic cells. Different lowercase letters represent significant differences between groups (*p* < 0.05); ns indicates not significant (*p* > 0.05).

The assessment of CLNE on cell viability was performed by CCK-8 assay. High cell viability implies low toxicity of the tested formulation. The results of the CCK-8 assay showed that the cell survival rates were more than 90% at concentrations ranging from 19.5 to 312 μg/mL after 24 h incubation (Figure 6A). Within this dosage range, the impact of the nanoemulsions on HOECs was minimal. The treatment group, with concentrations ranging from 39 to 156 μg/mL, showed no statistically significant differences compared to the control group. The cell survival rate in the 312 μg/mL was 104.61%, significantly higher than that in the other groups. This finding indicated that CLNE may have a stimulatory effect on cell proliferation at a certain concentration.

The effect of CLNE on the apoptosis of HOECs was further evaluated by flow cytometry with Annexin V-FITC/PI double staining. As shown in Figure 6B, the apoptosis rates for the treatment groups with concentrations of 156 and 312 μg/mL were 3.51% and 2.99%, whereas that of the control group was 2.70%. The late apoptosis/death rate was similar in the three groups with no significant difference (*p* > 0.05). Despite the slightly higher early and total apoptosis rates observed in the 156 μg/mL group compared to the control, the total apoptosis rate remained below 4%, suggesting minimal impact on cellular viability.

The essential oil derived from the congeneric plant *Cinnamomum burmannii* can enhance the proliferation of HaCaT cells within a specific concentration range [53]. In CLEO, 1,8-cineole has been demonstrated the potential to inhibit cell apoptosis and promote cell survival across different models. 1,8-cineole reduced the protein expression of bax, bim, cleaved caspase-3, and cleaved caspase-9 to alleviate glucose/amyloid-β (Aβ)-exposed human retinal pigment epithelial cell apoptosis [54]. 1,8-cineole suppressed isoproterenol (ISO)-induced apoptosis by regulating the ratio of Bax/Bcl-2 in H9c2 cardiomyocytes to improve heart function [55].

Based on these results, CLNE exhibited low toxicity towards oral epithelial cells at concentrations below 312 μg/mL, suggesting its potential application in broad fields within an appropriate dose.

## 4. Conclusions

In the present study, nanoemulsions of CLEO were successfully fabricated using the emulsifier EL-40. The mean droplet size of CLNE was 39.99 ± 0.47 nm and relatively narrow in distribution. The results of TEM imaging and centrifugation tests indicated that the nanoemulsions produced by the high-pressure homogenization method displayed better dispersion and stability compared to those prepared by the self-emulsifying method. In the industrial context, the use of the high-pressure method for the production of CLNE is considered a superior option. CLNE exhibited antibacterial activity against *E. coli* and *S. aureus*. The MIC and MBC values were lower than those of the pure oils. SEM observation of bacterial cell morphology and intracellular lysate leakage determinations revealed the antibacterial mechanism as the disruption of cell walls and the alteration of cell membrane permeability. The bacterial cell wall and membrane might be the target of CLEO. The reducing capacity of CLNE was higher than that of CLEO by DPPH and ABTS^•+^ radical scavenging assays. It can be deduced that the smaller particle size and improved dispersion in the medium of the nanoemulsions enhanced the various biological activities of the essential oils. At certain dosages, CLNE did not cause an excessive reduction in cell viability or an increase in apoptosis, and even at specific concentrations, they stimulated cell proliferation. Further research is needed to identify the components in CLEO that promote cell proliferation and their potential mechanisms. In conclusion, the formulations in this study provide theoretical support for expanding the application of CLEO in food preservation, feed additives, drug delivery, and daily chemical industries.

## Figures and Tables

**Table 1:** Chemical constituents of essential oils isolated from *C. longepaniculata* leaves.

No	Retention Time (min)	Compound	Molecular Formula	Relative Content (%)
1	11.34	α-Pinene	C_10_H_16_	7.40
2	12.9	Camphene	C_10_H_16_	0.23
3	14.74	β-Pinene	C_10_H_16_	4.28
4	15.4	Sabinene	C_10_H_16_	12.11
5	17.25	β-Myrcene	C_10_H_16_	1.74
6	17.56	α-Phellandrene	C_10_H_16_	0.27
7	18.45	α-Terpinene	C_10_H_16_	2.18
8	19.86	D-Limonene	C_10_H_16_	1.29
9	21.1	1,8-Cineole	C_10_H_18_O	49.49
10	22.82	γ-Terpinene	C_10_H_16_	2.74
11	24.53	p-Cymene	C_10_H_14_	0.63
12	25.52	α-Terpinolene	C_10_H_16_	0.90
13	25.9	Isoterpinolene	C_10_H_16_	0.09
14	34.17	trans-4-Thujanol	C_10_H_18_O	0.43
15	36.55	Camphor	C_10_H_16_O	0.39
16	37.2	Linalool	C_10_H_18_O	0.08
17	37.56	trans-β-Terpineol	C_10_H_18_O	0.25
18	38.12	trans-2-Menthenol	C_10_H_18_O	0.16
19	38.91	Bornyl acetate	C_12_H_20_O_2_	0.07
20	39.18	β-Elemene	C_15_H_24_	0.05
21	39.68	Terpinen-4-ol	C_10_H_18_O	3.82
22	40.05	Aromandendrene	C_15_H_24_	0.04
23	40.56	cis-2-Menthenol	C_10_H_18_O	0.11
24	41.51	Alloaromadendrene	C_15_H_24_	0.06
25	42.19	δ-Terpineol	C_10_H_18_O	0.81
26	42.39	α-Humulene	C_15_H_24_	0.49
27	43.18	α-Terpineol	C_10_H_18_O	7.70
28	43.4	Borneol	C_10_H_18_O	0.12
29	43.83	Germacrene D	C_15_H_24_	0.11
30	44.74	Bicyclogermacrene	C_15_H_24_	0.96
31	46.59	Nerol	C_10_H_18_O	0.10
		Monoterpenes		97.39
		Sesquiterpenes		1.71
		Total		99.1

**Table 2:** MIC and MIC of CLEO and CLNE against *E. coli* and *S. aureus*.

	CLEO		CLNE	
Bacteria	MIC (mg/mL)	MBC (mg/mL)	MIC (mg/mL)	MBC (mg/mL)
*Escherichia coli*	5	6	2	2
*Staphylococcus aureus*	7	8	3	5

## Data Availability

Data are contained within this article.
